# Goal-Oriented Care: A Catalyst for Person-Centred System Integration

**DOI:** 10.5334/ijic.5520

**Published:** 2020-11-04

**Authors:** Carolyn Steele Gray, Agnes Grudniewicz, Alana Armas, James Mold, Jennifer Im, Pauline Boeckxstaens

**Affiliations:** 1 Bridgepoint Collaboratory for Research and Innovation, Lunenfeld-Tanenbaum Research Institute, Sinai Health System, Toronto, ON, CA; 2 Institute of Health Policy, Management and Evaluation, University of Toronto, Toronto, ON, CA; 3 Telfer School of Management, University of Ottawa, Ottawa, ON, CA; 4 Department of Family and Preventive Medicine, College of Medicine, University of Oklahoma, US; 5 Community Health Centre Botermarkt, Ledeberg, BE; 6 Department of Public Health and Primary Care, Ghent University, BE

**Keywords:** people-driven care, goal-oriented care, integrated care, people-centred goals, case studies

## Abstract

**Introduction:**

Person-centred integrated care is often at odds with how current health care systems are structured, resulting in slower than expected uptake of the model worldwide. Adopting goal-oriented care, an approach which uses patient priorities, or goals, to drive what kinds of care are appropriate and how care is delivered, may offer a way to improve implementation.

**Description:**

This case report presents three international cases of community-based primary health care models in Ottawa (Canada), Vermont (USA) and Flanders (Belgium) that adopted goal-oriented care to stimulate clinical, professional, organizational and system integration. The Rainbow Model of Integrated Care is used to demonstrate how goal-oriented care drove integration at all levels.

**Discussion:**

The three cases demonstrate how goal-oriented care has the potential to catalyse integrated care. Exploration of these cases suggests that goal-oriented care can serve to activate formative and normative integration mechanisms; supporting processes that enable integrated care, while providing a framework for a shared philosophy of care.

**Lessons learned:**

By establishing a common vision and philosophy to drive shared processes, goal-oriented care can be a powerful tool to enable integrated care delivery. Offering plenty of opportunities for training in goal-oriented care within and across teams is essential to support this shift.

## Introduction

### Background

At the heart of the World Health Organization’s definition of integrated service delivery lies the aim that “integrated care should be centred on the needs of individuals, their families and communities” [1, p.4]. Person-centred care is not only aligned with integrated service delivery but has also been argued to be means to improve quality of care. In their 2001 report *Crossing the Quality Chasm*, the United States’ Institute of Medicine suggests that improving quality in care delivery requires a fundamental shift towards respecting and responding to patient preferences, needs and values [2]. Unfortunately, a person-centred approach to care is often at odds with how current health care systems are structured, as they take a problem-oriented view that seeks to treat illness and disease rather than looking at the individual within their context [3]. It is perhaps this tension that has slowed progress towards person-centred care delivery [4].

While person-centred care tends to be broadly defined so that implementation of the model can be adapted to meet local contextual needs [5], implementation challenges persist. Multiple studies point to factors at micro, meso and macro levels that can influence adoption of the model; including organizational and system structures, organizational culture, leadership approaches, as well as individual beliefs of providers regarding the value of the approach to care [6,7,8,9]. The University of Gothenburg Centre for Person-Centred Care in Sweden developed an evidence-based framework intended to support implementation of person-centred care [10,11]. The model focuses on how providers work one-on-one with their patients rather than supporting person-centred delivery by interprofessional and inter-organizational teams. As such, this may not be sufficient in cases where the aim is to implement person-centred care in integrated models that rely on care delivery by interprofessional and inter-organizational teams. This paper argues that, *goal-oriented care* (GOC) offers such an approach.

This case report describes the philosophy and activities of GOC, linking it to person-centred integrated care delivery. These connections are illustrated through the presentation of three international cases which have adopted (decided to commit to and use [12]) GOC as a means of putting person-centred and integrated care into practice.

### Goal-oriented care

The GOC concept was first introduced by Mold and colleagues in 1991 [13] as an alternative to problem-oriented care, particularly for individuals with diverse and complex health conditions and social care needs. In this introduction, GOC is viewed as conducive to team-based and interprofessional care (a cornerstone of integrated care) as it encourages team members to work together towards a common goal: that of the patient. More recently, GOC has gained momentum as an approach to tailoring and prioritizing care for multimorbid patients [14,15,16,17,18]. Drawing on the literature and an interdisciplinary clinical panel of experts, a clinically driven definition of GOC was published, defining it as:

*“the overarching aims of medical care for a patient that are informed by patients’ underlying values and priorities, established within the existing clinical context, and used to guide decisions about the use of or limitation on specific medical interventions”* [19, p.3]

This definition emphasizes using what is most important to the person seeking care to determine what kinds of care are appropriate and how care is to be delivered; which is, in essence, person-centred care.

Person-centred care delivery can be viewed as a paradigm shift requiring a change in the practice and teaching of medicine from one dominated by technical rationality to one that acknowledges the complex thoughts and interactions of patients and providers [20]. As one approach to person-centred care, GOC requires the same transformation. While clinicians are trained to use their clinical expertise to make care decisions to solve health problems, to be effective in a GOC approach, “clinicians must set aside their personal preferences and interests and listen actively to identify those [preferences] of their patients” [21]. Moving from a model that focuses on *problem-solving strategies* and *clinician-relevant objectives* towards addressing patient priorities effectively operationalizes a person-centred care approach and enables the four key components of people-driven care: empowerment and engagement, co-production and co-design.

First, GOC creates an environment which supports empowerment (gaining control over care) [22] and engagement [23] of individuals in their care, as the process requires that *individuals become active participants* in their own health, care and treatment. Setting goals can also improve patient self-efficacy needed to motivate action [24], like engaging in one’s care. Second, an important component of GOC in practice is the *co-production* and *co-design* of care plans between patients and their providers. Sanders and Strappers’ [25] foundational work on service co-design suggests that all participants be viewed as equal contributors to the creative process. GOC requires that patients and providers collaboratively create goals that can connect what is meaningful to both parties [16], requiring both collaboration and creativity to co-produce care goals used to guide the co-design of care plans.

Finally, co-production and co-design of care plans can translate to co-design of service delivery models. Given that people, particularly those with complex health and social care needs, will have diverse goals [21,26,27], existing siloed health care delivery models are often insufficient to meet care needs [28]. By adopting GOC at the practice level, service delivery gaps can be revealed, offering guidance on how to structure models to better meet the needs of patients. Here is where GOC can become a catalyst for implementing models of integrated health and social care.

### Goal-oriented care as a catalyst for integration

To best understand how GOC can drive adoption of person-centred integrated care, we connect the process and aims of GOC to Valentijn’s Rainbow Model of Integrated Care (RMIC) [29]. The RMIC suggests that six dimensions of integrated care need to be in place to achieve the model’s desired aims [30,31]. The RMIC dimensions were first identified via a systematic review of the literature [32] and later validated using a Delphi study and survey [33,34]. Table 1 presents how the dimensions of integrated care can be achieved through the adoption of a GOC approach.

**Table 1:** Mapping goal-oriented care to integrated care.

Rainbow Model of Integrated Care Dimensions	Definition [32]	Goal-Oriented Care Approach

*Micro level – clinical integration*	“The coordination of person-focused care in a single process across time, place and discipline”	GOC operationalizes a person-focused approach in a clinical encounter by calibrating care plans to person-identified goals and priorities, rather than working towards goals related to a specific disease, profession or setting.
*Meso level – professional integration*	“Interprofessional partnerships based on shared competencies, roles, responsibilities and accountability to deliver a comprehensive continuum of care to a defined population”	Goals are often diverse and complex, requiring support from different health and social care professionals. All team members need to understand and agree to focus on common goals (specifically, the patient’s), which can support the transcending of differences between disciplines and lead to clarification of roles and responsibilities in delivery of care.
*Meso level – organizational integration*	“Inter-organizational relationships (e.g. contracting, strategic alliances, knowledge networks, mergers) including common governance mechanisms, to deliver comprehensive services to a defined population”	Services required to meet diverse patient goals are likely to come from multiple organizations. Working towards common patient-prioritized goals can help establish a shared language and vision for professionals working together across organizational boundaries. Organizations can look beyond their siloed approaches to establish a shared vision and aligned governance structures.
*Macro level – system integration*	“A horizontal and vertical integrated system, based on a coherent set of (informal and formal) rules and policies between care providers and external stakeholders for the benefit of people and populations.”	When adopted across a wide region or network, GOC can be used to drive the structure of partnerships to better align with person-centred needs. For example, pay-for-performance systems need to attend to relevant and appropriate outcomes in order to be successful in integrated care [35]. Focusing too much on biomedical targets can have deleterious effects particularly for multi-morbid complex patients [36]. Goal-attainment has been argued to be a more appropriate outcome for multi-morbid patients [16].
**Mechanisms linking micro, meso and macro**		

*Functional integration*	“Key support functions and activities (i.e. financial, management and information systems) structured around the primary process of service delivery to coordinate and support accountability and decision-making between organisations and professionals in order to add overall value to the system.”	GOC creates a unifying process of care delivery that can inform the structure of coordinating activities (e.g., referral pathways) and information sharing (e.g., shared electronic medical records). For example, information sharing platforms can highlight person-centred goals, and indicate different providers and organizations that need to be involved in addressing the identified goals.
*Normative integration*	“The development and maintenance of a common frame of reference (i.e. shared mission, vision, values and culture) between organisations, professional groups and individuals.”	GOC can serve as a common philosophy, and a building block towards shared values of person-centeredness to align disparate professional and organizational groups that need to work together in an integrated model.

### Purpose

To illustrate the connections between GOC and integrated care proposed in Table 1, three international cases that have adopted the GOC model to drive clinical, professional, organizational and system level integration are presented. Case descriptions use data from three international case studies exploring the implementation of GOC models. Data to inform the comparative case study project was collected over the summer and fall of 2017 and included interviews with providers and managers and document reviews. Each case had a local lead researcher working with a trainee to collect data (Vermont lead – CSG; Ottawa lead – AG; Flanders lead – PB). Interviews were conducted with 18 providers and managers (18 in Vermont, 13 in Ottawa and 17 in Flanders for a total of 48). Data has and continues to be analysed to address core research questions of the study. What is presented here are overview case descriptions that are informed by these case studies to illustrate connections between GOC and integrated care, but should not be considered an in-depth exploration of the comparative case study data. Ethics approvals for the comparative research study were given by all relevant agencies in each of the three countries. This paper is a first step towards validation of the connections between GOC and integrated care, but is mainly intended to demonstrate how GOC can activate mechanisms that drive implementation of integrated health and social care delivery.

## International Cases of Goal-Oriented Care

### Clinical and professional integration in Ontario, Canada

GOC was introduced at a Community Health Centre (CHC) in Ontario, Canada through two programs aimed to improve care for patients with complex health and social needs. Ontario CHCs are publicly funded primary care organizations that provide care to vulnerable populations using an interprofessional approach. The CHC studied was organized into several teams, including a medical clinic, social services, a seniors outreach program, and the ‘Health Links’ program. The ‘Health Links’ program was a policy initiative launched by the Ontario Ministry of Health and Long-Term Care in 2012, which was intended to improve care coordination for ‘high service-use’ patient populations [37]. While person-centred care had always been part of the culture at the CHC, the specific GOC approach was implemented slowly over almost a decade and with the introduction of the seniors outreach program and the ‘Health Link’ program. As a result, GOC practice remained the leading approach only within these two programs, with minimal spread to the medical clinic and social services teams.

At the time of data collection, the seniors outreach program had been in place for almost 8 years, delivering services to vulnerable seniors at home. Registered nurses and community health workers would visit patients to perform in-home assessments, coordinate care with the patient’s primary care provider, provide education and social supports, and help the patient navigate the system through case management. The Health Links program was newer and implemented broadly across the province. The CHC led a voluntarily formed Health Links network (of various health and social organizations in the region) that aimed to work together to better coordinate care for patients with complex health and social needs. Given the overlap in their activities and aims, Health Links and the seniors outreach program were later integrated to expand seniors’ access to care coordination [38].

The main GOC tool at the CHC was the Health Links coordinated care plan which, among other details, outlined the patient’s goals. The care plans were completed with the patient, most often by a care coordinator, and then shared with the whole care team. The Health Links team documented all patient goals, even when unrealistic, and the patient’s goals always took precedent over clinicians’ goals when there was disagreement. This approach was taken due to the patient-centeredness philosophy at the CHC. When goals were not achievable, they were viewed as a guide for the patient’s ideal care plan. When used by providers in practice, the care plan created clinical integration by bringing the care team together (e.g., physician, social worker, addictions counsellor) to provide care that aligned with stated patient goals. Care coordinators and other team members would meet regularly to discuss their most challenging cases and the team would work to identify solutions in line with the patient’s goals using local resources, for example moving a patient to safer housing. The seniors outreach program also provided goal-directed case management.

Members of the CHC saw the GOC approach as an opportunity to create professional integration. Though there was no explicit push for GOC in the medical clinic or social services teams, it appeared that there was some diffusion of the approach through the Health Links and seniors outreach programs. However, this was only successful where professionals on a patient’s care team all ‘bought in’ to GOC and the use of the care plan. The teams faced additional challenges when trying to use a GOC approach with the patient’s care providers that were *outside* of the organization and were either not familiar with care plans or had not bought in to the GOC approach. However, the CHC was building momentum within its walls, with multiple professionals (social workers, family doctors, care coordinators) using the patient’s explicit goals to guide care. While not all CHC providers used GOC in practice, they all subscribed to the philosophy behind GOC and its alignment with the person-centered culture at the CHC.

### Professional and organizational integration in Vermont, USA

In 2009, the state of Vermont in the United States underwent a major health care funding transformation. Driven by a desire to better serve “high-needs” Medicare and Medicaid clients, the Blueprint for Health initiative was established to lead the transformation of primary and comprehensive health services funding and delivery. One of the objectives of Blueprint for Health was to support community-led programs by deploying quality improvement practice facilitators to work with local health and social care providers to design and deliver new models of care. Different models were introduced, including Community Health Teams, Patient-Centred Medical Homes, Hub and Spoke models, and special population supports (e.g., The Women’s Health Initiative) [39].

One rural Community Health Team program adopted a GOC approach to support coordination of care across health and social care services. The Community Health Team included providers from multiple health and social care agencies delivering services in the region including primary care physicians, care coordinators, behavioural health specialists, home care providers, mental health support providers, and a case manager situated in the local hospital. Among the challenges facing the practice facilitator was how to support professional and organizational integration; bringing together disparate groups of providers who each had their own professional backgrounds and training, organizational knowledge and culture, and different clinical and professional skills and competencies.

The practice facilitator introduced the GOC approach, building on a set of tools from the Camden Coalition for Healthcare Providers which seeks to improve care delivery for individuals with complex health and social care needs [40]. The Camden Coalition has created a set of tools (Camden Domain Cards and Eco Mapping) to help elicit individual’s goals which are then used to develop care plans; a method they call “backwards planning” [41]. Using these tools, the practice facilitator developed a 2-day training session for providers joining the Community Health Team, offering the training multiple times as needed for providers to learn about GOC and practice using the tools.

After training was complete, members of the Community Health Team met bi-weekly in-person to discuss complex cases that required support from the different team members. Providers would present cases for discussion, often the provider with the closest relationship to the individual, or the one who first identified the individual as a good candidate to be treated by the Community Health Team. In discussing and troubleshooting cases providers often referred back to the individual’s goals, identified when the patient was first brought into the program. In conducting the case study, researchers had an opportunity to observe a meeting and were struck by how frequently the patient goals were mentioned, used to redirect the conversation towards what mattered to the patient, and guided assigning of responsibilities of different members of the team to support the patient.

In interviews with the 18 providers and managers working in the model, conducted about 18 months after the start of the program, all providers defined GOC in the same way as “meeting people where they’re at”, suggesting a coalescing of beliefs regarding patient care. Providers and managers indicated that by setting their own clinical goals aside and focusing instead on patient goals, they were better able to work as an interprofessional team. Interprofessional team-work was reinforced through bi-weekly meetings where providers developed a better understanding of each of their skills supporting role clarity, established shared responsibility, and built relationships necessary to support professional-level integration.

At an organizational level, focusing on client goals enabled identification of which organizations and providers needed to be involved in the Community Health Team to provide necessary ‘wrap around’ services to clients. For this case, these organizations included the local hospital, 3 primary care centres, community seniors care agency, housing support, and a mental health agency. One gap identified in the interviews was lower than hoped primary care physician engagement (only one actively participated in the group). The team managed this challenge by having more active involvement by a primary care administrator and establishing behavioural health therapists and nurses in primary care clinics to liaise between the Community Health Team and physicians as needed. Organizational managers and regional leaders saw GOC as a useful way to identify how funding models and agreements needed to be modified to integrate care across organizations. At the time of data collection, the region was considering updating the information infrastructure (e.g., adopting an electronic shared care plan) to improve communication between providers and client access to services. An information system was also viewed as a mechanism to enable future system-level integration by identifying population health needs and trends.

### From clinical to organisational to system integration in Flanders, Belgium

In Flanders, Belgium patients, providers and policy makers have created momentum for GOC through numerous initiatives at clinical, organizational and system levels. First, since 2010, two CHCs in Ghent introduced GOC explicitly while improving and reworking their processes of interprofessional collaboration for patients with chronic care needs. In both CHCs, teams were looking to enhance role clarity across disciplines to improve efficiency and support interdisciplinary collaboration and overall care. Interprofessional care plans were developed for patients with complex care needs that were guided by patient goals. Goals were established through in-depth conversations between that patient and their provider with whom they the closest therapeutic relationship (often a nurse), and informal caregivers where appropriate. Goals were aligned to the International Classification of Functioning [42]. Care teams would then hold a 60–90 minute meeting (preferably with the patient) to integrated all the information into a care plan which was then added to the patient’s electronic medical record and used as a reference to organize interprofessional care; supporting clinical integration for the patient within the CHC. Similar to the Vermont example, interprofessional team meetings were guided by a GOC approach, creating a common language and approach that facilitated professional integration.

A second initiative led by the Flemish government, was the introduction of local multidisciplinary networks to support primary care providers to implement chronic disease management programs. In 2013, one of these networks, Regio Gent, started local interprofessional training meetings where professionals from different disciplines gathered to learn about disease-specific topics such as diabetes or chronic obstructive pulmonary disease. In 2015, Regio Gent decided to shift their focus from a disease-oriented approach to a community focus. Through a partnership with Ghent University GOC was identified as a promising approach to guide local interprofessional meetings intended to advance community collaboration. Primary care networks included professionals within a specific community who collaborated and shared accountability for the health and well-being of all the people living in their community. Interprofessional community meetings originally mainly gathered primary care professionals such as physicians, nurses, physiotherapists and dieticians but over time increasingly engaged social workers and professionals working in homecare, contributing to professional integration.

Regio Gent also engaged with Maastricht University, University College Zuyd and a consultancy agency (Dubois & Van Rij) to provide two-day training on person-centered care to help introduce GOC to emerging primary care networks. At the time of writing, over 130 professionals had been trained, and the local 90-minute meetings were still being organized using a train-the-trainer approach.

A third initiative occurring at the regional level in Flanders started in 2010 with primary care reform efforts aimed at restructuring the system. As part of these efforts, the Flanders region was divided into 60 primary care zones serving geographically defined areas of 100,000–150,000 citizens. Primary care zones were (and continue to be) governed by a care council representing health, social and community sector stakeholders, and include representatives from professional organizations (representing primary care clinicians, patients, and informal carers), homecare institutions (e.g. retirement homes and home care services), and community supports (e.g. centres for welfare, social security, health insurance, mental health supports, and child preventive services). The zones are supported by the Flemish Institute for Primary Care in partnership with Ghent University to develop a train-the-trainer program and strategy. Among strategies is advancing the implementation of GOC. One of these primary care zones explicitly defined GOC as a building block for care in their vision and mission statement in 2018.

Lastly, in 2018, GOC was introduced as one of the potential strategies for proactive and person-centered primary care for the population experiencing moderately complex problems by the Primary Care Academy. The Academy is a research and teaching network of four Flemish universities, six university colleges, a home nursing organization (the White-Yellow Cross) and patient representatives which promote and study GOC as an innovative strategy for proactive and person-centered care in interprofessional models. In close collaboration with different stakeholders at the micro, meso and macro level, the Academy aims to develop roadmaps and hands-on toolkits for primary care policies, practice and education, using GOC as a means to advance person-centered integrated care from a system level.

## Discussion

### Goal-oriented care catalysing micro, meso and macro integration

The three presented cases demonstrate how GOC was a catalyst for integration at micro, meso and macro levels. Across all three cases, GOC was used to support clinical integration by bringing together different clinical professionals to coordinate care delivery based on patient-identified goals. Cases used tools like care plans (in Ottawa and Flanders) or goal-elicitation processes (like the Camden Cards used in Vermont) to bring different clinicians onto the same page in terms of care delivery. Patients and caregivers were involved in the creation of care plans based on patient goals, speaking to the ability for GOC to support *co-production* of care delivery. Creating this space for co-production at the clinical level has been shown to be an important stepping stone towards more patient involvement in co-designing system and organizational changes [43], suggesting adopting GOC may also support wider system *co-design* with patients and caregivers. However, patient and family involvement in care tended to focus at the stage of goal elicitation, and did not always translate to ongoing engagement, necessary to support person-centred care processes [44].

The presented cases demonstrate how GOC enabled professional and organizational level integration at the meso level. This was particularly evident in the Vermont and Flanders cases where integrated care delivery spanned organizational boundaries. In these instances, GOC was used to establish communication pathways, support role clarity at the professional level, and identify and create partnerships required to provide patients with access to the breadth of services needed to address their goals. This type of shared understanding has been found to be important for effective interprofessional teamwork [45]. In Flanders, partnerships not only spanned health and social care organizations but included regional decision-making bodies and universities as a means to support evidence-based training in the model and set the foundation for broader system level change. Physician engagement, however, was a challenge in the Ottawa and Vermont cases, which is consistent with previous research in this area [46,47,48].

The value of GOC to support system level integration was still in the stage of creating a common vision at the time of data collection for these three cases. However, Flanders, and to a lesser extent Vermont, were using GOC as a key driver of reform efforts intended to improve integrated care delivery at a system level. Their vision included attention to structures, agreements and funding models that could be aligned using GOC, however time will tell if GOC will help to make this system-level vision a reality. For example, Flanders 2025 primary care reform strategic priorities includes an aim to establish care teams the support individuals “in accordance with his or her life goals” [49, p.23].

### Goal-oriented care enabling formative and normative mechanisms of integration

Perhaps the greatest learning from these three cases comes from looking at how GOC activates the *mechanisms* that drive integrated care. In the three cases we see examples of how GOC was able to activate formative integration mechanisms, by offering a process by which clinicians could work together within and across their organizations. Specifically, GOC asks clinicians and managers to focus on the thing they have in common, regardless of their professional or organizational backgrounds, that is – the goals of their patients. This focus streamlines communication and processes, as seen in the Vermont example where patient goals were used to keep the large interprofessional meetings on track and centred on finding solutions to better serve patients.

Maybe most important is how GOC was able to activate normative integration, which was key when working between different organizations that had different cultures and driving visions. In the presented cases GOC helped create a framework to establish a shared vision amongst providers and organizations, identified as necessary to drive integrated care models [50,51]. As noted earlier, GOC, like person-centred care, requires a philosophical shift in how providers, managers, and patients think about the delivery of health care services. For those clinicians and teams who had already “bought in” to the ethos, such as the two programs in the Ottawa case, GOC was used mainly formatively, to drive processes. But in cases that worked with “outsiders”, or across different organizations, as was the case in Vermont and Flanders, more intensive training was used to get groups aligned to this ethos of care, arguably activating normative integration of values across diverse professional and organizational groups. Of course, there are many additional factors that drive organizational change. A deeper exploration of the training processes and how these can drive a change in beliefs, as well as how they interrelate with other drivers like engagement and leadership buy-in found to influence organizational uptake of person-centred care [52] would provide a more thorough understanding of organizational change.

What the cases do demonstrate is the power of using GOC as an approach to get the many different players involved in integrated health and social care delivery aligned to a common vision; delivering care that addresses what is important to patients. Or as the providers would say in Vermont, to “meet them where they’re at.”

## Lessons Learned

The three case examples presented offer several lessons for those seeking to adopt GOC as a driver for their integration efforts.

1. To drive *clinical integration*, health and social care providers, as well as patients and their informal caregivers, need to be trained in how to engage and be engaged in a GOC approach and be given opportunities to put the model into practice. Tools like those described in the cases can be used to support GOC at the clinical level.
2. To drive *professional integration*, inter- and intra-organizational processes need to be established where professionals can easily share information and communicate around person-identified goals.
3. To drive *organizational integration*, person-centred goals can be used to create shared aims and vision across disparate organizations, which should be strongly supported and reinforced by senior leadership.
4. To drive *system integration*, GOC can be used to create a common vision and road map for system level reform efforts towards greater vertical and horizontal integration, as was the case in Flanders.
5. To drive *formative integration*, organizations and regions need to invest in resources and tools (e.g., information systems) that enable a shared GOC approach within and across the health and social care systems.
6. To drive *normative integration*, intensive training in the approach and philosophy of GOC should be regularly available for clinicians, managers and patients. Preferably training should be done together to reinforce shared beliefs and values related to the model of care.

## Conclusions

The international cases of GOC presented in this case report serve to demonstrate how GOC can be used to catalyse implementation of person-centred integrated care models. Using the Rainbow Model, the cases show that GOC can serve to activate formative and normative mechanisms to drive integration at clinical, professional and organizational levels, with a view towards broader system level integration. The next step requires that proposed links between GOC and integrated care be tested using empirical data. Co-authors are exploring opportunities to develop a survey tool that could be deployed across the members of the Goal-Oriented Care Learning Collaborative. The Collaborative was founded by the co-authors of this paper, and is an international network of researchers, providers and health system leaders seeking to advance the adoption of GOC in their regions. Through advancing GOC internationally, there is an opportunity to help systems move away from problem-oriented thinking towards whole-person and community-based models that are better positioned to deliver person-centred and integrated models of care.

## References

[1] Integrated care models: An overview. Copenhagen, Denmark: World Health Organization; 2016 [cited 2020 7 Feb]. https://www.euro.who.int/en/health-topics/Health-systems/health-services-delivery/publications/2016/integrated-care-models-an-overview-2016.

[2] Institute of Medicine (US) Committee on Quality of Health Care in America. Crossing the Quality Chasm: A New Health System for the 21st Century Washington, DC: National Academies Press (US); 2001 DOI: 10.17226/10027 25057539

[3] Mold J. Goal-directed health care: Redefining health and health care in the era of value-based care. Cureus. 2017; 9(2): e1043 DOI: 10.7759/cureus.1043 28367382 PMC5360382

[4] Ahmad N, Ellins J, Krelle H, Lawrie M. Person-centred care: From ideas to action London, UK: Health Foundation; 2014.

[5] Naldemirci Ö, Wolf A, Elam M, Lydhal D, Moore L, Britten N. Deliberate and emergent strategies for implementing person-centred care: A qualitative interview study with researchers, professionals and patients. BMC Health Services Research. 2017; 17: 527 DOI: 10.1186/s12913-017-2470-2 28778167 PMC5545038

[6] Carlström E, Ekman I. Organisational culture and change: Implementing person-centred care. Journal of Health Organization and Management. 2012; 26(2): 175–91. DOI: 10.1108/14777261211230763 22856175

[7] Hower K, Vennedey V, Hillen H, Kuntz L, Stock S, Pfaff H, et al. Implementation of patient-centred care: which organisational determinants matter from decision maker’s perspective? Results from a qualitative interview study across various health and social care organisations. BMJ Open. 2019; 9(4): e027591 DOI: 10.1136/bmjopen-2018-027591 PMC6500213 30940764

[8] Moore L, Britten N, Lydhal D, Naldemirci Ö, Elam M, Wolf A. Barriers and facilitators to the implementation of person-centred care in different healthcare contexts. Scandinvian Journal of Caring Sciences. 2016; 31(4): 662–73. DOI: 10.1111/scs.12376 PMC5724704 27859459

[9] Mork Rokstad A, Vatne S, Engedal K, Selbaek G. The role of leadership in the implementation of person-centred care using Dementia Care Mapping: A study in three nursing homes. Journal of Nursing Management. 2013; 23(1): 15–26. DOI: 10.1111/jonm.12072 23678892

[10] Ekman I, Hedman H, Swedberg K, Wallengren C, Göteborgs universitet, Centrum för personcentrerad vård vid Göteborgs universitet (GPCC), et al. Commentary: Swedish initiative on person-centred care. BMJ. 2015; 350: h160 DOI: 10.1136/bmj.h160 25670185

[11] Ekman I, Swedberg K, Taft C, Lindseth A, Norberg A, Brink A, et al. Person-centred care – Ready for prime time. European Journal of Cardiovascular Nursing. 2011; 10: 248–51. DOI: 10.1016/j.ejcnurse.2011.06.008 21764386

[12] Rabin B, Brownson R, Haire-Joshu D, Kreuter M, Weaver N. A Glossary for Disseminaton and Implementation Research in Health. Journal of Public Health Management and Practice. 2008; 14(2): 117–23. DOI: 10.1097/01.PHH.0000311888.06252.bb 18287916

[13] Mold J, Blake G, Becker L. Goal-oriented medical care. Family Medicine. 1991; 23(1): 46–51. 2001782

[14] Allen K, Hazelett S, Radwany S, Ertle D, Fosnight S, Moore P. The Promoting Effective Advance Care for Elders (PEACE) randomized pilot study: Theoretical framework and study design. Population Health Management. 2012; 15(2): 71–7. DOI: 10.1089/pop.2011.0004 22088165

[15] Boyd C, Boult C, Shadmi E, Leff B, Brager R, Dunbar L, et al. Guided care for multimorbid older adults. The Gerontologist. 2007; 47(5): 697–704. DOI: 10.1093/geront/47.5.697 17989412

[16] Reuben D, Tinetti M. Goal-oriented patient care – An alternative health outcomes paradigm. New England Journal of Medicine. 2012; 366(9): 777–9. DOI: 10.1056/NEJMp1113631 22375966

[17] Tinetti M, Aanand N, Dodson J. Moving from disease-centered to patient goals-directed care for patients with multiple chronic conditions: Patient value-based care. JAMA Cardiology. 2016; 1(1): 9–10. DOI: 10.1001/jamacardio.2015.0248 27437646 PMC6995667

[18] Upshur C, Weinrib L. A survey of primary care provider attitudes and behaviors regarding treatment of adult depression: What changes after a collaborative care intervention? Primary Care Companion Journal of Clinical Psychiatry. 2008; 10(3): 182–6. DOI: 10.4088/PCC.v10n0301 PMC2446486 18615167

[19] Secunda K, Wirpsa M, Neely K, Szmuilowicz E, Wood G, Panozzo E, et al. Use and Meaning of “Goals of Care” in the Healthcare Literature: a Systematic Review and Qualitative Discourse Analysis. Journal of general internal medicine. 2019; 35(5): 1559–1566. DOI: 10.1007/s11606-019-05446-0 31637653 PMC7210326

[20] Reach G. Simplistic and complex thought in medicine: The rationale for a person-centred care model as a medical revolution. Patient Preference and Adherence. 2016; 10: 449–57. DOI: 10.2147/PPA.S103007 27103790 PMC4829191

[21] Goal-oriented care In: Bensadon B (ed.), Psychology and Geriatrics. 1 ed: Academic Press; 2015 p. 3 DOI: 10.1016/B978-0-12-420123-1.00001-0

[22] World Health Organization. Health Promotion Glossary Geneva: World Health Organization; 1998 [cited 2020 7 February]. Available from: https://www.who.int/healthpromotion/about/HPR%20Glossary%201998.pdf?ua.

[23] Gallivan J, Kovacs Burns K, Bellows M, Eigenseher C. The many faces of patient engagement. Journal of Participatory Medicine. 2012; 12 26; 4: e32

[24] Bandura A. Self-efficacy: Toward a unifying theory of behavioral change. Psychological Review. 1977; 84(2): 191–215. DOI: 10.1037/0033-295X.84.2.191 847061

[25] Sanders E, Stappers P. Co-creation and the new landscapes of design. CoDesign. 2008; 4(1): 5–18. DOI: 10.1080/15710880701875068

[26] Mold J. An alternative conceptualization of health and health care: It’s implications for geriatrics and gerontology. Educational Gerontology. 1995; 21(1): 85–101. DOI: 10.1080/0360127950210109

[27] Steele Gray C, Gravesande J, Kaur Hans P, Nie J, Sharpe S, Loganathan M, et al. Using exploratory trials to identify relevant contexts and mechanisms in complex electronic health interventions: Evaluating the Electronic Patient-Reported Outcome tool. JMIR Formative Research. 2019; 3(1). DOI: 10.2196/11950 PMC6414821 30810532

[28] van der Aa M, van den Broeke J, Stronks K, Plochg T. Patients with multimorbidity and their experiences with the healthcare process: A scoping review. Journal of Comorbidity. 2017; 7(1): 11–21. DOI: 10.15256/joc.2017.7.97 29090185 PMC5556434

[29] Valentijn P. Rainbow of chaos: A study into the theory and practice of integrated primary care. International Journal of Integrated Care. 2016; 16(2). DOI: 10.5334/ijic.2465

[30] Institute for Healthcare Improvement. IHI Triple Aim Initiative: Institute for Healthcare Improvement; 2020 [cited 2020 7 February]. Available from: http://www.ihi.org/Engage/Initiatives/TripleAim/Pages/default.aspx.

[31] Greaves F, Pappas Y, Bardsley M, Harris M, Curry N, Holder H, et al. Evaluation of complex integrated care programmes: the approach in North West London. International Journal of Integrated Care. 2013; 13: e006 DOI: 10.5334/ijic.974 23687478 PMC3653284

[32] Valentijn P, Boesveld I, van der Klauw D, Ruwaard D, Struijs J, Molema J, et al. Towards a taxonomy for integrated care: A mixed-methods study. International Journal of Integrated Care. 2015; 15(1). DOI: 10.5334/ijic.1513 PMC4353214 25759607

[33] Valentijn P, Angus L, Boesveld I, Nurjono M, Ruwaard D, Vrijhoef H. Validating the Rainbow Model of Integrated Care Measurement Tool: Results from three pilot studies in the Netherlands, Singapore and Australia. International Journal of Integrated Care. 2017; 17(3). DOI: 10.5334/ijic.3203

[34] Valentijn P, Pereira F, Sterner C, Vrijhoef H, Ruwaard D, Hegbrant J, et al. Validation of the Rainbow Model of Integrated Care Measurement Tools (RMIC-MTs) in renal care for patient and care providers. PLoS ONE. 2019; 14(9): e0222593 DOI: 10.1371/journal.pone.0222593 31536548 PMC6752779

[35] Tsiachristas A. Payment and economic evaluation of integrated care. International Journal of Integrated Care. 2015; 15: e013 DOI: 10.5334/ijic.2009 26034470 PMC4447214

[36] Boyd C, Darer J, Boult C, Fried L, Boult L, Wu A. Clinical practice guidelines and quality of care for older patients with multiple comorbid diseases: Implications for pay and performance. JAMA. 2005; 294(6): 716–24. DOI: 10.1001/jama.294.6.716 16091574

[37] Grudniewicz A, Tenbensel T, Evans JM, Steele Gray C, Baker GR, Wodchis WP. ‘Complexity-compatible’ policy for integrated care? Lessons from the implementation of Ontario’s Health Links. Social science & medicine (1982). 2018; 198: 95–102. DOI: 10.1016/j.socscimed.2017.12.029 29310110

[38] Austin T, Chreim S, Grudniewicz A. Examining health care providers’ and middle-level managers’ readiness for change: a qualitative study. BMC Health Services Research. 2020; 20(47). DOI: 10.1186/s12913-020-4897-0 PMC6969476 31952525

[39] State of Vermont. About the Blueprint Vermont: State of Vermont; 2019 [cited 2020 7 February]. Available from: https://blueprintforhealth.vermont.gov/about-blueprint.

[40] Camden Coalition. Camden coalition of healthcare providers: Camden Coalition; 2020 [cited 2020 7 February]. Available from: https://camdenhealth.org/.

[41] Camden Coalition. Create a backwards plan: Instructional guide New Jersey: Camden Coalition; 2016 [cited 2020 7 February]. Available from: https://camdenhealth.org/wp-content/uploads/2016/12/Backwards-Planning_Instructional-Guide-Board-Domain-Cards.pdf.

[42] World Health Organization. International classification of functioning, disability and health (ICF): World Health Organization; 2018 [cited 2020 7 February]. Available from: https://www.who.int/classifications/icf/en/.

[43] Turakhia P, Combs B. Using principles of co-production to improve patient care and enhance value. AMA J Ethics. 2017; 19(11): 1125–31. DOI: 10.1001/journalofethics.2017.19.11.pfor1-1711 29168684

[44] Santana MJ, Manalili K, Jolley RJ, Zelinsky S, Quan H, Lu M. How to practice person-centred care: A conceptual framework. Health expectations: an international journal of public participation in health care and health policy. 2018; 21(2): 429–40. DOI: 10.1111/hex.12640 29151269 PMC5867327

[45] Franklin CM, Bernhardt JM, Lopez RP, Long-Middleton ER, Davis S. Interprofessional Teamwork and Collaboration Between Community Health Workers and Healthcare Teams: An Integrative Review. Health Serv Res Manag Epidemiol. 2015; 2: 2333392815573312-. DOI: 10.1177/2333392815573312 PMC5266454 28462254

[46] Béland F, Bergman H, Lebel P, Clarfield AM, Tousignant P, Contandriopoulos A-P, et al. A System of Integrated Care for Older Persons With Disabilities in Canada: Results From a Randomized Controlled Trial. The Journals of Gerontology: Series A. 2006; 61(4): 367–73. DOI: 10.1093/gerona/61.4.367 16611703

[47] Curry N, Harris M, Gunn LH, Pappas Y, Blunt I, Soljak M, et al. Integrated care pilot in north-west London: a mixed methods evaluation. Int J Integr Care. 2013; 13: e027 DOI: 10.5334/ijic.1149 24167455 PMC3807631

[48] Embuldeniya G, Kirst M, Walker K, Wodchis WP. The Generation of Integration: The Early Experience of Implementing Bundled Care in Ontario, Canada. Milbank Quarterly. 2018; 96(4): 782–813. DOI: 10.1111/1468-0009.12357 30417941 PMC6287073

[49] WHO Regional Office for Europe. Creating 21st century primary care in Flanders and beyond Copenhagen, Denmark; 2019. Contract No.: Licence: CC BY-NC-SA 3.0 IGO.

[50] Goodwin N. Taking integrated care forward: the need for shared values. International Journal of Integrated Care. 2013; 13: e026 DOI: 10.5334/ijic.1180 23882173 PMC3718261

[51] Zonneveld N, Raab J, Minkman MMN. Towards a values framework for integrated health services: an international Delphi study. BMC health services research. 2020; 20(1): 224 DOI: 10.1186/s12913-020-5008-y 32183785 PMC7079447

[52] Bokhour B, Fix G, Mueller N, Barker A, Lavela S, Hill J, et al. How can healthcare organizations implement patient-centered care? Examining a large-scale cultural transformation. BMC Health Services Research. 2018; 18(168). DOI: 10.1186/s12913-018-2949-5 PMC5842617 29514631

